# Supplementary material for: Multiplexed histology analyses for the phenotypic and spatial characterization of human innate lymphoid cells
Source: Nat Commun. 2021 Mar 19;12:1737. doi: 10.1038/s41467-021-21994-8 (PMC7979823; doi:10.1038/s41467-021-21994-8)
Supplement: Supplementary file 1 — Supplementary Information [file 41467_2021_21994_MOESM1_ESM.pdf]

Supplementary Information

Multiplexed histology analyses for the phenotypic and spatial characterization of human innate lymphoid cells

Anna Pascual-Reguant, Ralf Köhler, Ronja Mothes, Sandy Bauherr, Daniela Hernández, Ralf Uecker, Karolin Holzwarth, Katja Kotsch, Maximilian Seidl, Lars Philipsen, Werner Müller, Chiara Romagnani, Raluca Niesner and Anja E. Hauser

Correspondence to Anja E. Hauser, anja.hauser-hankeln@charite.de, hauser@drfz.de

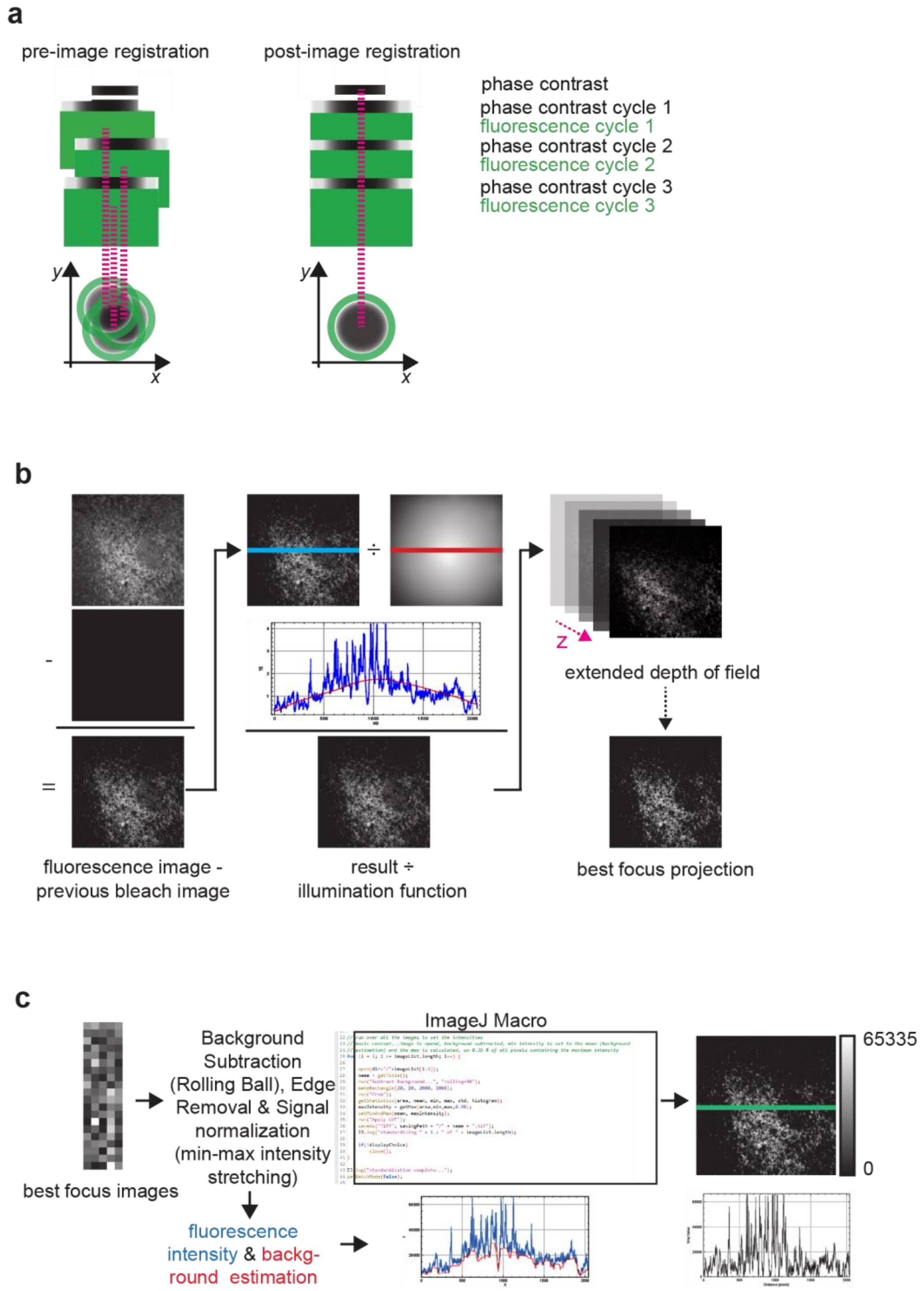

Supplementary Fig. 1. Image pre-processing. (a) In each cycle of the MELC Run, phase contrast images and fluorescent images are acquired. Phase contrast images are used for XY alignment of all fluorescent images in order to correct minimal XY shifts due to mechanical perturbations. This image registration procedure is performed by cross-correlation with a reference phase contrast image of 512 x 512 pixels acquired at the very beginning of the measurement. **(b)** Registered MELC images are further pre-processed: first, by subtracting the bleaching image of the previous MELC cycle; second by correcting for illumination artifacts, based on the mean normalized bleaching image of the current cycle and, finally, by an *Extended*

Depth of Field algorithm that corrects differences in z focusing and creates an *all in focus* projection. **(c)** Image normalization macro in ImageJ/Fiji for further data processing. Background Subtraction, based on a *Rolling ball* algorithm followed by edge removal, where edge width is taken from the maximum allowed shift in the autofocus procedure. Finally, intensity signal is stretched to the full intensity range (16 bit -> 2^{16}).

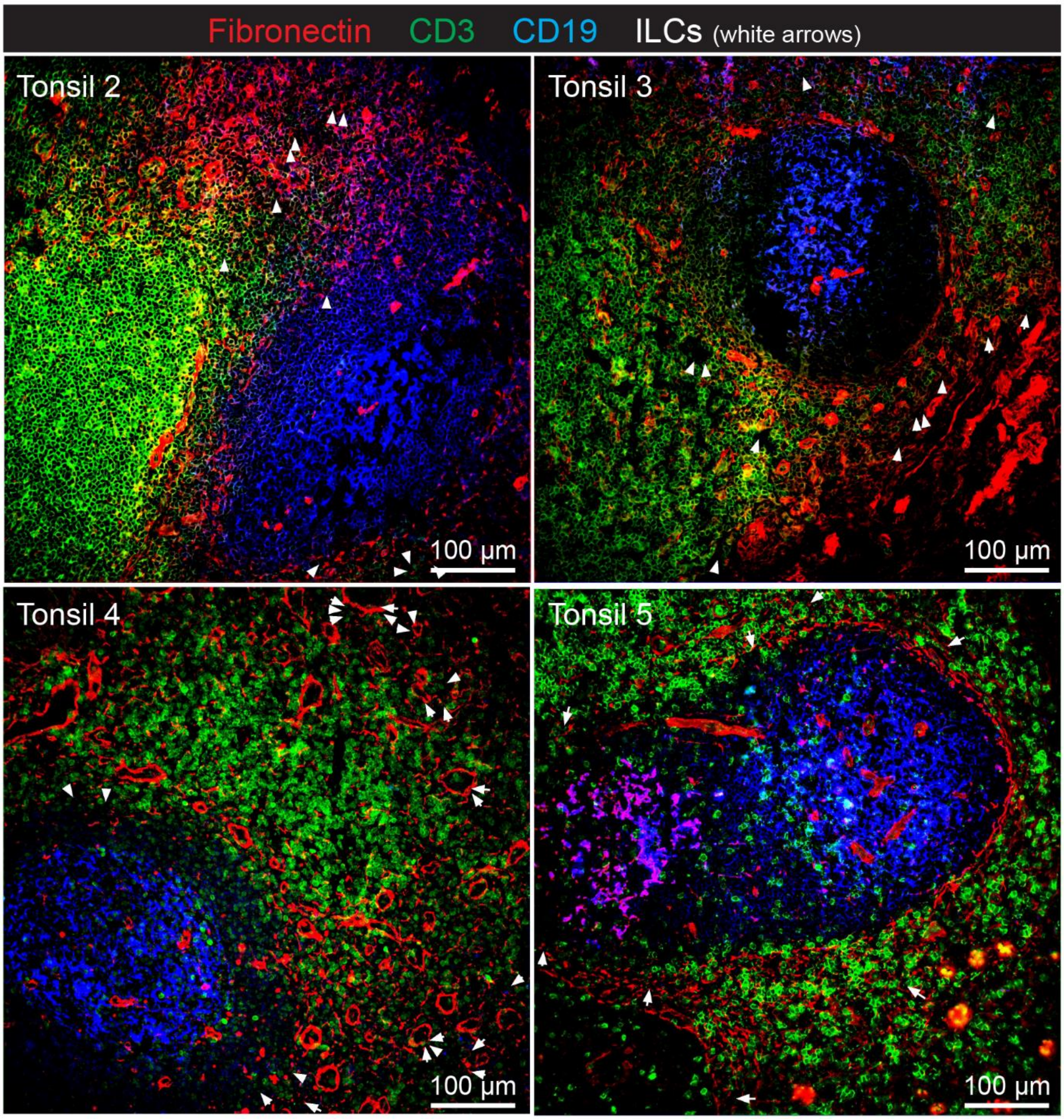

Supplementary Fig. 2. Tonsillar ILCs show a particular localization pattern in relation to stromal landmarks. Structural overview of the tonsil from 4 different samples, where B cell follicles, T cell zone and fibronectin-rich areas are shown by staining of CD19 (blue), CD3 (green) and Fibronectin (red), respectively. ILCs are shown as white arrows and are localized in tight association with fibronectin fibers, but not within the B cell follicles and rarely deep within the T cell zone. (n = 5).

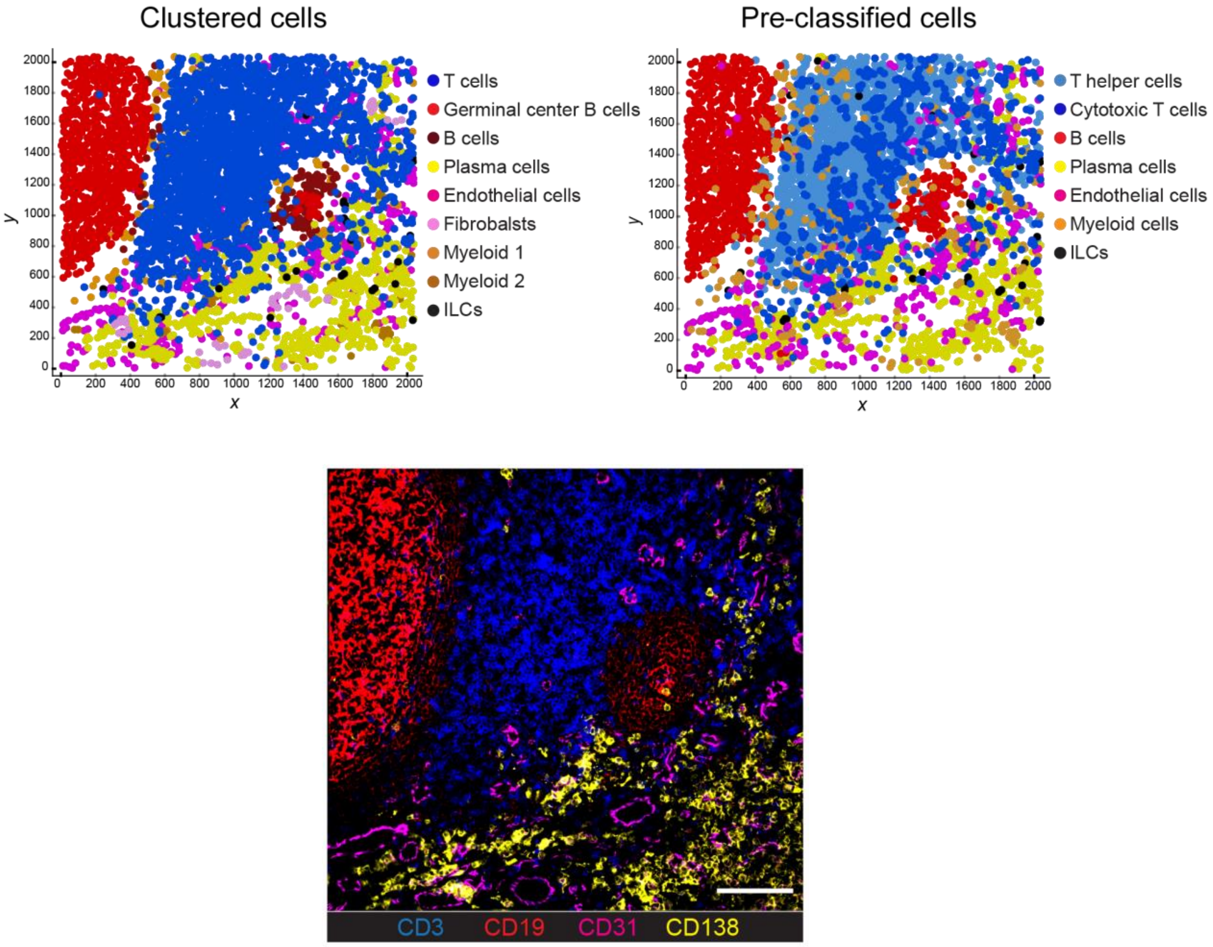

Supplementary Fig. 3. Tonsillar ILCs and plasma cells accumulate in the highly vascularized connective tissue septum. Upper panel: allocation of color-coded clustered cells (T cells in blue, germinal center B cells in red, B cells in dark brown, plasma cells in yellow, endothelial cells in magenta, fibroblasts in pink, monocytes in orange, macrophages in light brown and ILCs in black) and pre-classified cells (T helper cells in light blue, cytotoxic T cells in dark blue, B cells in red, plasma cells in yellow, endothelial cells in magenta, myeloid cells in orange and ILCs in black) within spatial coordinates, in which dots represent nuclear centroids. Bottom panel: staining of $CD19^+$ B cells (red), $CD3^+$ T cells (blue), $CD31^+$ endothelial cells (magenta) and $CD138^+$ plasma cells (yellow) shown as overlay of fluorescence images. Scale bar: 100 µm. (n = 5).

a

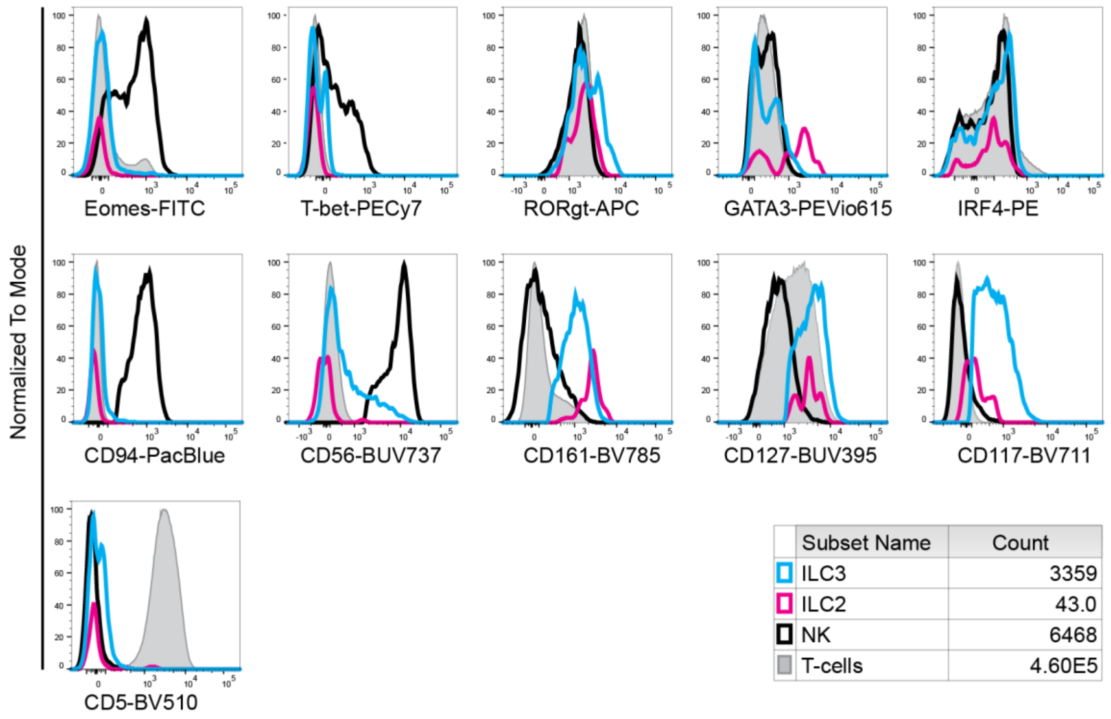

b

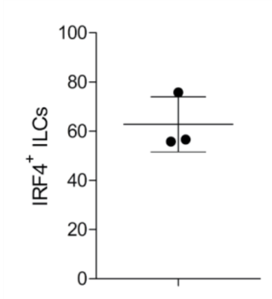

Supplementary Fig. 4. Flow cytometry analysis confirms IRF4 expression in tonsillar ILC3. (a) Histograms depict expression of the intracellular transcription factors Eomes, T-bet, RORγt, GATA3 and IRF4, as well as the surface markers CD94, CD56, CD161, CD127, CD117 and CD5 in T-cells (grey), NK cells (black outline), ILC2 (magenta outline), ILC3 (cyan outline) as gated in Fig. 7d. **(b)** Dot plot showing the frequency of IRF4^{+} ILCs, as gated in Fig. 7d. Data are shown as mean ± S.D. (n = 3). Source data are provided as a Source Data file.

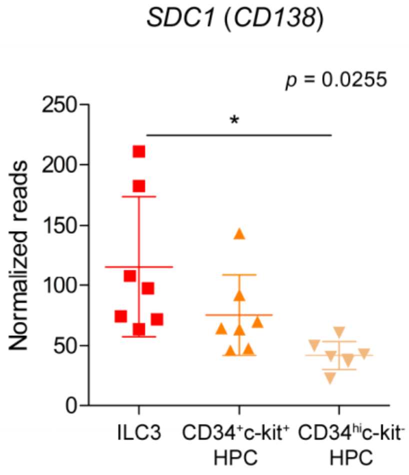

Supplementary Fig. 5. *CD138* is expressed in ILC3 at the transcriptional level. Dot plot depicting *SDC1* (*CD138*) normalized transcript reads in sorted DAPI$^-$Lin$^-$CD94$^-$CD127^{hi}CD56$^+$ ILC3s, CD34$^+$c-Kit$^+$ hematopoietic progenitor cells (HPC) and CD34^{hi}c-Kit$^-$ HPC, extracted from published data [40]. Data shown as mean ± S.D analyzed by one-way ANOVA (F= 5.062 and 2 *df*) with Bonferroni´s multiple comparison test, where * $p < 0.05$ (n=7). Source data are provided as a Source Data file.

ObjectNumber	CD127	CD138	CD14	CD141	CD16	CD161	CD19	CD3	CD4	CD45	CD49a	CD56	CD68	CD69	cell_class
**810**	0,088	0,026	7,79E-04	0,003	0,014	0,34	7,50E-04	0	0	0,026	0,014	0,012	0,149	0,014	ILC
**811**	0,021	0,348	0,01	0,007	0,034	0,005	0,028	0,004	0	0,038	5,45E-04	0,006	0,129	0,019	Plasma cell
**812**	8,70E-04	0,046	0,002	7,03E-04	0,022	0,003	0	2,92E-04	0	0,006	0	0,003	0,005	0	Plasma cell
**813**	0,014	0	0,002	0,001	0,054	8,93E-04	0	0	0	0,007	0,004	0,023	0,104	0	other
**814**	0,027	0,018	7,52E-04	0,04	0,011	6,49E-04	0	0,037	2,50E-04	0,023	0,066	0,007	0,104	0,008	other
**815**	0,055	0,044	0	0,017	0,006	0,003	3,03E-04	0,032	0	0	0,028	0,005	0,065	0,003	Plasma cell
**830**	0,338	3,35E-04	4,54E-04	0,05	0,02	0,302	0	0,295	0,145	0,081	0,084	0,013	0,102	0,018	T helper cell
**831**	0,007	0,003	0	4,83E-04	0,008	0,038	0	0	0	0	0	0,001	0,002	0	ILC
**848**	0,225	0,085	3,09E-05	0,002	0,016	0,558	0	5,38E-04	0	9,46E-05	0,015	0,005	0,005	0,014	ILC
**2752**	0,082	7,92E-04	0,04	0,211	0,038	0,007	0	0,36	0,032	0,063	0,098	0,06	0,022	0,006	T cytotoxic cell
**2753**	0,136	0,241	0,004	0,198	0,08	0,003	0,022	0,084	0,018	0,018	0,067	0,014	0,188	9,58E-04	T cytotoxic cell
**2754**	0,024	0	0,1	0,019	0,009	2,16E-04	0,024	0,042	0,037	0,092	0	0,023	0,061	0,161	other
**2755**	0,117	0	0,002	0,017	0,01	0,27	8,43E-06	0,17	0,39	0,196	0,003	0,026	0,037	0,116	T helper cell
**2756**	0,129	1,06E-04	0,017	0,103	0,042	0,041	0	0,099	0,35	0,192	0,123	0,265	0,167	0,026	T helper cell
**2771**	0,029	0	0,008	0,003	0,51	0,01	0,001	0,031	0,022	0,032	0,008	0,012	0,008	0,001	other
**2772**	0,012	0,02	0,193	0,009	0,023	0,003	0,298	0,008	0,035	0,065	0,002	0,046	0,202	0,064	B cell
**2773**	0,079	0	0,004	0,082	0,03	0,103	0	0,14	0,263	0,163	0,07	0,239	0,116	0,124	T helper cell
**2774**	0,145	0	0,026	0,016	0,039	0,456	0	0,238	0,397	0,173	0,001	0,031	0,053	0,374	T helper cell
**2775**	0,117	0	0,006	0,023	0,022	0,003	0	0,345	0,022	0,012	0,034	0,015	0,131	0,006	T cytotoxic cell
**2776**	0,124	0,371	0,016	0,037	0,083	0,174	0,006	0,054	0,061	0,046	0,04	0,07	0,263	0,063	T helper cell
**2777**	0,167	0,143	0,017	0,017	0,084	0,05	0,018	0,003	0,069	0,082	0,024	0,044	0,313	0,053	Plasma cell
**2790**	0,136	3,29E-05	0,062	0,085	0,034	0,133	1,58E-04	0,083	0,226	0,094	0,112	0,159	0,124	0,106	T helper cell
**2791**	0,335	0	0,054	0,01	0,039	0,265	1,48E-04	0,338	0,363	0,111	7,56E-04	0,013	0,061	0,066	T helper cell
**2792**	0,119	0	0,023	0,077	0,102	0,013	0,01	0,06	0,083	0,051	0,189	0,056	0,231	0,026	T helper cell
**2793**	0,119	0,079	0,002	0,031	0,007	0,005	0,002	0,181	0,011	0,031	0,006	0,006	0,05	1,68E-04	T cytotoxic cell
**2794**	0,056	0	0,001	0,003	0,055	0,007	1,35E-04	0,068	0	0,02	0,034	5,76E-04	0,027	0	T cytotoxic cell
**2795**	0,044	0,031	0,247	0,012	0,037	0,009	0,051	0,266	0,063	0,136	0	0,013	0,089	0,667	B cell
**2796**	0,025	6,23E-05	0,129	0,019	0,025	0,001	0,049	0	0	0,007	0	0,008	0,064	0,032	B cell
**2803**	0,047	6,13E-04	0,022	0,013	0,038	0,003	0,073	0	0	0,144	0,009	0,017	0,085	0,022	B cell
**2816**	0,092	0	0,082	0,642	0,033	0,021	3,41E-04	7,47E-04	0,01	0,043	0,493	0,259	0,157	0,025	Monocyte/Macrohage/DC
**2817**	0,259	0	1,36E-04	0,003	0,05	0,473	1,13E-04	0,015	0,001	0,042	0,003	0,041	0,074	0,15	ILC
**2818**	0,07	0	0,138	0,163	0,027	0,009	8,54E-04	0,018	0,015	0,058	0,258	0,106	0,087	0,023	Monocyte/Macrohage/DC

Supplementary Table 1. Excerpt of the data table from CellProfiler. Analysis performed in CellProfiler is extracted as a data table containing object numbers (left column) and annotated cell types (right column), as well as the corresponding MFI of every marker for all objects (exemplarily shown for CD127, CD138, etc.; middle columns). Additionally, the corresponding center X and Y coordinates are also preserved (not shown).

Antibody	Fluorochrome	Clone	Company	Dilution
DAPI			Roche	1:5000
Fibronectin		rabbit IgG	Invitrogen	1:200
anti-rabbit	PE	Goat IgG	Rockland	1:200
CD127	PE	REA279	Miltenyi	1:50
Collagen IV	FITC	2F11	AntibodiesOnline	1:500
ICOS	PE	REA192	Miltenyi	1:50
Ki67	FITC	MIB1	Dako	1:50
Bcl6	PE	REA373	Miltenyi	1:10
SMA	FITC	1A4	Abcam	1:100
KLRG1	PE	REA261	Miltenyi	1:10
IRF4	PE	REA201	Miltenyi	1:10
FcER1a	PE	CRA1	Miltenyi	1:10
Foxp3	PE	PCH101	Invitrogen	1:50
NKp44	PE	2.29	Miltenyi	1:10
Pax5	PE	REA140	Miltenyi	1:11
RANKL	PE	DN254	Miltenyi	1:10
Helios	PE	22F6	Biolegend	1:50
CD123	PE	AC145	Miltenyi	1:10
Eomes	PE	WD1928	BD BioScience	1:50
CD161	PE	191B8	Miltenyi	1:10
CD16	PE	REA423	Miltenyi	1:10
CD138	PE	M115	Biolegend	1:50
CD14	PE	Tük4	Miltenyi	1:10
CD141	PE	BDCA-3	Miltenyi	1:10
CD19	PE	LT19	Miltenyi	1:10
CXCR3	PE	REA232	Miltenyi	1:10
c-Kit	PE	A3C6E2	Miltenyi	1:10
CD103	PE	Ber-ACT 8	Miltenyi	1:10
CD49a	PE	TS2/7	Biolegend	1:50
VCAM-1	PE	REA269	Miltenyi	1:10
CCR4	PE	REA279	Miltenyi	1:10
CD56	PE	AF12-7H3	Miltenyi	1:10
CD11c	PE	MJ4-27G12	Miltenyi	1:10
CD69	PE	REA824	Miltenyi	1:50
CCR5	PE	J418F1	Biolegend	1:50
CD94	PE	REA113	Miltenyi	1:10
IL1R1	PE	REA744	Miltenyi	1:10
CCR6	PE	REA190	Miltenyi	1:10
Granzyme A	PE	REA162	Miltenyi	1:10
IgA	PE	REA995	Miltenyi	1:10
CD200R	PE	OX-108	Biolegend	1:50
CD31	PE	9G11	R&D	1:50
IgG	PE	IS11-3B2 23	Miltenyi	1:10
Langerin	PE	REA770	Miltenyi	1:100
TCRγδ	PE	REA591	Miltenyi	1:50
IgM	PE	PJ2-22H3	Miltenyi	1:50
CD21	PE	REA940	Miltenyi	1:50
CD23	PE	M-L23.4	Miltenyi	1:10
PD1	PE	REA1165	Miltenyi	1:50
CD34	PE	AC136	Miltenyi	1:10
CD38	PE	IB6	Miltenyi	1:10
CD45RA	PE	REA562	Miltenyi	1:10
CD163	PE	RM3/1	Biolegend	1:50
CD45RO	PE	UCHL1	DRFZ	1:50
TCRVα7.2	PE	mouse 3C10	Biolegend	1:50
CD4	PE	VIT4	Miltenyi	1:50
CD20	PE	LT20	Miltenyi	1:10
CD8	PE	BW135/80	Miltenyi	1:50
HLA-DR	PE	REA332	Miltenyi	1:10
CD3	PE	REA613	Miltenyi	1:50
CD45	PE	5B1	Miltenyi	1:50
CD7	PE	CD7-6B7	Miltenyi	1:50
Vimentin	A488	EPR3776	Abcam	1:200

Supplementary Table 2. MELC antibody panel.

Antigen	Fluorochrome	Clone	Company	Dilution
CD117	BV711	104D2	Biolegend	1:50
CD123	APC-eF780	6H6	eBioscience	1:50
CD127	BUV395	HIL-7R-M21	BD	1:200
CD14	APC-eF780	61D3	eBioscience	1:50
CD141	APC-Vio770	REA674	Miltenyi	1:50
CD161	BV785	HP-3G10	Biolegend	1:50
CD19	APC-eF780	HIB19	eBioscience	1:50
CD20	APC-Vio770	LT20	Miltenyi	1:50
CD3	BUV805	SK7	BD	1:50
CD45	AF700	HI30	Biolegend	1:50
CD5	BV510	L17F12	Biolegend	1:100
CD56	BUV737	NCAM16.2	BD	1:50
CD94	PacB	XA185	In House	1:100
CRTH2	PerCP Cy5.5	BM16	Biolegend	1:10
Eomes	Fitc	WD1928	eBioscience	1:20
FceRIa	APC-Vio770	CRA1	Miltenyi	1:50
GATA3	PE-Vio615	REA174	Miltenyi	1:20
IRF4	PE	REA201	Miltenyi	1:50
LD	APCeF780		eBioscience	1:500
NKp44	Biotin	P44-8	Biolegend	1:400
RORyt	APC	REA278	Miltenyi	1:10
Streptavidin	BUV496		BD	1:200
T-bet	PE-Cy7	4B10	Biolegend	1:400

Supplementary Table 3. Flow cytometry antibody panel.
