# Supplementary material for: Multiplexed histology analyses for the phenotypic and spatial characterization of human innate lymphoid cells
Source: Nat Commun. 2021 Mar 19;12:1737. doi: 10.1038/s41467-021-21994-8 (PMC7979823; doi:10.1038/s41467-021-21994-8)
Supplement: Supplementary file 3 — Reporting Summary [file 41467_2021_21994_MOESM3_ESM.pdf]

nature research

Corresponding author(s): Anja E. Hauser

Last updated by author(s): Feb 19, 2021

Reporting Summary

Nature Research wishes to improve the reproducibility of the work that we publish. This form provides structure for consistency and transparency in reporting. For further information on Nature Research policies, see our Editorial Policies and the Editorial Policy Checklist.

Statistics

For all statistical analyses, confirm that the following items are present in the figure legend, table legend, main text, or Methods section.

n/a	Confirmed	
☐	☒	The exact sample size (*n*) for each experimental group/condition, given as a discrete number and unit of measurement
☐	☒	A statement on whether measurements were taken from distinct samples or whether the same sample was measured repeatedly
☐	☒	The statistical test(s) used AND whether they are one- or two-sided<br>*Only common tests should be described solely by name; describe more complex techniques in the Methods section.*
☒	☐	A description of all covariates tested
☒	☐	A description of any assumptions or corrections, such as tests of normality and adjustment for multiple comparisons
☐	☒	A full description of the statistical parameters including central tendency (e.g. means) or other basic estimates (e.g. regression coefficient) AND variation (e.g. standard deviation) or associated estimates of uncertainty (e.g. confidence intervals)
☐	☒	For null hypothesis testing, the test statistic (e.g. *F*, *t*, *r*) with confidence intervals, effect sizes, degrees of freedom and *P* value noted<br>*Give P values as exact values whenever suitable.*
☒	☐	For Bayesian analysis, information on the choice of priors and Markov chain Monte Carlo settings
☒	☐	For hierarchical and complex designs, identification of the appropriate level for tests and full reporting of outcomes
☒	☐	Estimates of effect sizes (e.g. Cohen's *d*, Pearson's *r*), indicating how they were calculated

Our web collection on statistics for biologists contains articles on many of the points above.

Software and code

Policy information about availability of computer code

Data collection	The software TIC-Control (Version 5.4) was used to control the pipetting robot and the microscope system used for data acquisition.
Data analysis	ImageJ 1.52i was used to pre-process and normalize fluorescence images.<br>Ilastik 1.3.2 was used to perform pixel classification (pixels were classified into nuclei, membranes and extracellular compartment).<br>CellProfiler 3.1.8 was used for cell segmentation, for extracting single-cell features (mean fluorescence intensities per cell), for cell classification and for neighborhood analysis.<br>Orange 3.26 was used for clustering analysis of single-cell data and for neighborhood analysis of the colon data set.<br>FlowJo v.10.6.01 software (TreeStar) was used for the analysis of flow cytometry data.<br><br>All software used for data acquisition and data analysis has already been published elsewhere and no new code has been written.

For manuscripts utilizing custom algorithms or software that are central to the research but not yet described in published literature, software must be made available to editors and reviewers. We strongly encourage code deposition in a community repository (e.g. GitHub). See the Nature Research guidelines for submitting code & software for further information.

Data

Policy information about availability of data

All manuscripts must include a data availability statement. This statement should provide the following information, where applicable:

- Accession codes, unique identifiers, or web links for publicly available datasets
- A list of figures that have associated raw data
- A description of any restrictions on data availability

All raw data (images) and materials (Cell Profiler pipelines) associated with this study are publicly available on the Zenodo open-access repository https://

zenodo.org/. The tonsil data-set under DOI 10.5281/zenodo.3744152 and the colon data-set under DOI 10.5281/zenodo.3744173. Complete and detailed CellProfiler pipelines and all data tables generated are publicly available under DOIs 10.5281/zenodo.3744206 and 10.5281/zenodo.3744273.
The raw data as well as log2 transformed normalized values for the microarray data are available under the GEO accession number GSE63197.

Field-specific reporting

Please select the one below that is the best fit for your research. If you are not sure, read the appropriate sections before making your selection.

- [x] Life sciences
- [ ] Behavioural & social sciences
- [ ] Ecological, evolutionary & environmental sciences

For a reference copy of the document with all sections, see nature.com/documents/nr-reporting-summary-flat.pdf

Life sciences study design

All studies must disclose on these points even when the disclosure is negative.

Sample size	For the in situ characterization of ILC in the tonsil, five independent experiments were performed yielding similar results in terms of ILC numbers, phenotypes and localization patterns and, therefore, n=5 was considered sufficient. For the in situ characterization of ILC in the colon, two independent experiments were performed yielding similar results in terms of ILC numbers, phenotypes and localization patterns. Since the localization patterns were additionally similar to those find in the tonsil, n=2 for the analysis of ILC in the colon was considered sufficient to highlight that ILC show conserved tissue landmarks.
Data exclusions	CCR4, CD121b, CD25, CD68, CRTH2, Granzyme B, KLRG1, NKp30, and NKp80 stainings were also included in the five tonsil MELC runs performed for this study. However, the image data and the single-cell data extracted from these were excluded from the final analysis because stainings were not consistent in one or more of the replicates.
Replication	For the analysis of ILC in tonsils, five independent experiments were performed and one representative example was shown. For the analysis of ILC in colon, two independent experiments were performed and one representative example was shown. All attempts at replication were successful for the experimental findings shown in the manuscript.
Randomization	n/a, because we do not compare different experimental groups
Blinding	n/a, because we do not compare different experimental groups

Reporting for specific materials, systems and methods

We require information from authors about some types of materials, experimental systems and methods used in many studies. Here, indicate whether each material, system or method listed is relevant to your study. If you are not sure if a list item applies to your research, read the appropriate section before selecting a response.

Materials & experimental systems

n/a	Involved in the study	
[ ]	[x]	Antibodies
[x]	[ ]	Eukaryotic cell lines
[x]	[ ]	Palaeontology and archaeology
[x]	[ ]	Animals and other organisms
[ ]	[x]	Human research participants
[x]	[ ]	Clinical data
[x]	[ ]	Dual use research of concern

Methods

n/a	Involved in the study	
[x]	[ ]	ChIP-seq
[ ]	[x]	Flow cytometry
[x]	[ ]	MRI-based neuroimaging

Antibodies

Antibodies used

For MELC:
DAPI (Roche), Fibronectin (rabbit IgG; Invitrogen), anti-rabbit-PE (donkey IgG, Rockland), CD127-PE (REA279; Miltenyi), COL-IV-FITC (2F11; AntibodiesOnline), ICOS-PE (REA192; Miltenyi), Ki67-FITC (MIB1; Dako), Bcl6-PE (REA373; Miltenyi), SMA-FITC (1A4; Abcam), KLRG1-PE (REA261; Miltenyi), IRF4-PE (REA201; Miltenyi), FcER1a-PE (CRA1; Miltenyi), FoxP3-PE (PCH101; Invitrogen), NKP44-PE (2.29; Miltenyi), Pax5-PE (REA140; Miltenyi), RANKL-PE (DN254 Miltenyi), Helios-PE (22F6; Biolegend), CD123-PE (AC145; Miltenyi), Eomes-PE (WD1928; BD BioScience), CD161-PE (191B8; Miltenyi), CD16-PE (REA423; Miltenyi), CD138-PE (M115; Biolegend), CD14-PE (Tük4; Miltenyi), CD141-PE (BDCA-3; Miltenyi), CD19-PE (LT19; Miltenyi), CXCR3-PE (REA232; Miltenyi), c-Kit-PE (A3C6E2; Miltenyi), CD103-PE (Ber-ACT 8; Miltenyi;), CD49a-PE (TS2/7; Biolegend), VCAM-1-PE (REA269; Miltenyi), CCR4-PE(REA279; Miltenyi), CD56-PE (AF12-7H3; Miltenyi), CD11c-PE (MJ4-27G12; Miltenyi), CD69-PE (REA824; Miltenyi), CCR5-PE (J418F1; Biolegend), CD94-PE (REA113; Miltenyi), IL1R1-PE (REA744; Miltenyi), CCR6-PE (REA190; Miltenyi), GranzA-PE (REA162; Miltenyi), IgA-PE (REA995; Miltenyi), CD200R-PE (OX-108; Biolegend), CD31-PE (9G11; R&D), IgG-PE (IS11-3B2 23; Miltenyi), Langerin-PE (REA770; Miltenyi), TCRγδ-PE (REA591; Miltenyi), IgM-PE (PJ2-22H3; Miltenyi), CD21-PE (REA940; Miltenyi), CD23-PE (M-L23.4; Miltenyi), PD1-PE (REA1165; Miltenyi), CD34-PE (AC136; Miltenyi), CD38-PE (IB6; Miltenyi), CD45RA-PE (REA562; Miltenyi), CD163-PE (RM3/1;

	Biolegend), CD45RO-PE (UCHL1; in house), TCRVα7.2-PE (3C10; Biolegend), CD4-PE (VIT4; Miltenyi), CD20-PE (LT20; Miltenyi), CD8-PE (BW135/80; Miltenyi), HLA-DR-PE (REA332; Miltenyi), CD3-PE (REA613; Miltenyi), CD45-PE (5B1; Miltenyi), CD7-PE (CD7-6B7; Miltenyi), Vimentin-A488 (EPR3776; Abcam)<br><br>For FACS:<br>CD117-BV711 (104D2; Biolegend), CD123-APC-eF780 (6H6; eBioscience), CD127-BUV395 (HIL-7R-M21; BD), CD14-APC-eF780 (61D3; eBioscience), CD141-APC-Vio770 (REA674; Miltenyi), CD161-BV785 (HP-3G10; Biolegend), CD19-APC-eF780 (HIB19; eBioscience), CD20-APC-Vio770 (LT20; Miltenyi), CD3-BUV805 (SK7; BD), CD45-AF700 (HI30; Biolegend), CD5-BV510 (L17F12; Biolegend), CD56-BUV737 (NCAM16.2; BD), CD94-PacB (XA185; in house), CRTH2-PerCP-Cy5.5 (BM16; Biolegend), Eomes-Fitc (WD1928; eBioscience), FceRIa-APC-Vio770 (CRA1; Miltenyi), GATA3-PE-Vio615 (REA174; Miltenyi), IRF4-PE (REA201; Miltenyi), LD-APC-eF780 (eBioscience), NKp44-Biotin (P44-8; Biolegend), RORyt-APC (REA278; Miltenyi), Streptavidin-BUV496 (BD), T-bet-PE-Cy7 (4B10; Biolegend)
Validation	All antibodies are commercially available and have been validated for flow cytometry and/or immunofluorescence by the manufacturers. We have titrated all antibodies for immunofluorescence and tested them all in several human tissues (including skin, thymus, SI, colon, kidney, LN, liver, cervix, tonsil and lung) assessing by visual inspection the pattern of expression in the tissues, expression level, sub-cellular distribution and co-localization with other lineage-defining markers, etc.

Human research participants

Policy information about studies involving human research participants

Population characteristics	Tonsil 1 (shown as representative example):<br>F, 26 Y<br>Diagnosis: recurrent tonsillitis.<br>No further clinical or genotypic information.<br><br>We have no information on gender, age, treatment, or any other feature from the other four tonsillectomy samples, as they were anonymous.<br><br>Colitis Ulcerosa 1 (shown as representative example):<br>M, 36 Y<br>Past diagnosis: colitis ulcerosa, refractory to therapy, colectomy and terminal ileostoma 02/2019. Current treatment: open residual protectomy w/ mucosectomy w/ ileostom. pouch + protect. Ileostoma<br>No secondary diagnosis<br>Intercurrent diagnosis: compartment syndrome<br>No medication at admission, pain medication upon need at dismissal<br><br>Colitis Ulcerosa 2:<br>M, 25 Y<br>Past diagnosis: colectomy 2018 + terminal ileostoma<br>Current treatment: pouch + residual protectomy<br>No secondary/ intercurrent diagnosis<br>No medication at admission, 3 day pain medication at dismissal
Recruitment	Tonsil 1 (shown as representative example): the patient was recruited as an immunologically healthy control after written informed consent, which has been reviewed and approved by the ethics committee of the Albert-Ludwigs-University of Freiburg, Germany. The sample used for the scientific purpose was taken after diagnostic sampling and exclusion of malignancy. Palatine tonsils as the chosed one are lymphoid organs with hyperplasia of mainly secondary lymphoid follicles, namely a B-zone expansion suggestive for a strong humoral activity. Therefore, it cannot be excluded that other lymphoid organs (e.g. lymph nodes) with interfollicular / paracortical hyerplasia, which speaks for a T-zone expansion, may display different spatial relations of ILCs with resident cells of the adaptive immune response.
Ethics oversight	Approval by the medical ethics commission of the University of Freiburg (251/13_140389) and the ethics commission of the Charité-Universitätsmedizin Berlin (EA2/078/16) were obtained, in accordance with the local ethical guidelines.

Note that full information on the approval of the study protocol must also be provided in the manuscript.

Flow Cytometry

Plots

Confirm that:

- [x] The axis labels state the marker and fluorochrome used (e.g. CD4-FITC).
- [x] The axis scales are clearly visible. Include numbers along axes only for bottom left plot of group (a 'group' is an analysis of identical markers).
- [x] All plots are contour plots with outliers or pseudocolor plots.
- [x] A numerical value for number of cells or percentage (with statistics) is provided.

Methodology

Sample preparation	Tonsils were thoroughly dissociated in sterile PBS/BSA/2mM EDTA and passed through a 70µm strainer. Mononuclear cell suspensions were isolated by Ficoll density gradient centrifugation and MACS-depletion of CD19+ and CD3+ cells (Miltenyi Biotec) was performed prior staining. Single cell suspension of tonsil mononuclear cells were stained for surface markers in 100µl PBS/BSA for 15 min at 37°C. After washing, biotin was stained with fluorochrome labelled streptavidin for 10 min at 4° C. Afterwards, cells were fixed for 35 min at RT, permeabilized and transcription factors (TF) were stained at RT for 45 min using the Foxp3 / Transcription Factor Staining Buffer Set (eBioscience).
Instrument	FACSymphony (BD Bioscience)
Software	FlowJo v.10.6.01 software (TreeStar)
Cell population abundance	Cell sorting techniques were not used in this study. Tonsil CD3+ and CD19+ B cells were depleted through MACS and remaining cells were identified and excluded through surface CD3 and CD19 staining. Populations were described by known markers and the abundance of populations is expressed as frequencies, as illustrated in gating strategies.
Gating strategy	Viable ILC gating in the tonsil: Initial lymphocyte population was gated by FSC-A vs SSC-A. Cells were then gated by FSC-A versus FSC-H, where any cells which did not express equal area and height were determined as doublets and excluded. The remaining cell population was then gated as shown in Figure 6, on CD45 expressing, lineage negative cells. Next, T lymphocytes expressing CD3 were excluded. NK cells were identified as CD94+ CD56+ cells which were excluded from subsequent gating. ILCs were identified as CD161+CD127+ cells. All gating was based on bimodality of marker expression.

☒ Tick this box to confirm that a figure exemplifying the gating strategy is provided in the Supplementary Information.
